# Assessment of gender differences in some inflammatory cytokines of tuberculosis patients before and during treatment

**DOI:** 10.4314/ahs.v23i3.40

**Published:** 2023-09

**Authors:** Chizoba Okeke, Robert Okonkwo, Nancy Ibeh, Oluchukwu Chukwuma, Chisom Okeke

**Affiliations:** 1 Nnamdi Azikiwe University; 2 Nnamdi Azikiwe University Teaching Hospital

**Keywords:** Tuberculosis, anti-tuberculosis drugs, cytokines, inflammation

## Abstract

**Background:**

Gender variation is a feature of many physiological parameters including inflammatory cytokines. Inflammation is an obvious feature of Tuberculosis (TB) infection with changes in pro and anti-inflammatory cytokines.

**Objective:**

To compare the levels of inflammatory cytokines between male and female TB patients before treatment, after 2-months and after 6-months anti-tuberculosis treatment.

**Materials and methods:**

A total of 35 males and 25 females TB subjects were enlisted before initiation of therapy and followed up after 2-months and 6 months treatment and samples collected and analysed. Tumour necrosis factor-alpha (TNF-α), Interleukin 10 (IL-10, Interleukin -6 (IL-6), Interleukin-2 (IL-2), transforming growth factor-beta (TGF-β) were assayed by ELISA method.

**Results:**

Before treatment, the median level of IL-6 (pg/ml) was significantly higher in males compared to female TB patients (P=0.046). While after 2-months treatment, TNF-α (pg/ml) and IL-10 (pg/ml) was significantly higher in males compared with females (P=0.008 and 0.045 respectively). Conversely, the median IL-6 (pg/ml) was significantly higher in female TB patients compared to the males (P=0.042). No significant differences were observed after 6-months treatment.

**Conclusion:**

Gender differences exist in IL-6 before treatment and in IL-6, TNF-α and IL-10 at two months treatment. Thus, TB treatment contributes differentially to levels of inflammatory cytokines in male and female TB patients.

## Introduction

Infectious diseases affect males and females unequally,^1^ and tuberculosis infection is not an exception as globally far more men than women have tuberculosis. Reports have shown that this sex differential in prevalence rates appears between 10 to 16 years of age and remain higher in males than females afterwards. This was also corroborated by the WHO Global TB report 2 that reported that out of the globally documented ten million people (range, 9.0–11.1 million) that developed TB disease in 2017; 5.8 million were men, 3.2 million women and one million were children. Although the cause of this bias is uncertain and the reason for the higher male prevalence and incidence is poorly understood, epidemiological factors have historically been considered the driving force.^3^ Shepherd and Alasdair 4 posits that gender can affect M. tuberculosis exposure because of differences in social roles, risk behaviours, and activities. Males may travel more frequently, have more social contacts, spend more time in settings that may be conducive for transmission, such as bars, and engage in professions associated with a higher risk for tuberculosis, such as mining.^5^

Males have been shown to be generally more susceptible to infectious diseases than females and when they are infected they tend to show a higher level of mortality.^6^ But in conversely, autoimmune diseases show more prevalence in females.^7^ According to Klingstrom *et al.*^8^ these gender variations in susceptibility to infectious diseases and in level of mortality between male and females probably arise from gender-related abilities to initiate proper immune responses against the invading infectious agent. Immunological differences between males and females can arise from diverse causes which may include hormonal, genetic, and microbiome differences between the two sexes.^9^

Cytokines are mediators of inflammation that play very important roles in maintaining the immune responses against infections. Elevated levels of some cytokines have been observed in various infectious diseases. Since gender differences influences the severity and evolution of various inflammatory conditions 10 one important area of likely sex differences is in the cytokine response evoked during infection. Currently, there has been conflicting findings on gender differences on the levels of inflammatory cytokines in different disease states. While some studies reported elevated cytokine production in men compared with women, others found the opposite while some found no difference between both sexes. 9-13

Several mechanisms have been advanced to explain the sex differences observed in inflammatory markers. Mendelsohn and Karas 14 proposed that female sex exerts a permissive influence over inflammatory responses, a phenomenon thought to be principally driven by the activity of female sex hormones, while Fish 15 asserted that as a general rule, females exhibit more-robust immune responses to antigenic challenges, such as infection and vaccination, than males which is mediated to a large extent by sex hormones. However, Casimir *et al* 11 agreed that gender influences clinical presentations and markers in inflammatory diseases, but contested the notion of hormonal involvement in the gender difference in inflammatory markers and opined that the persistence of this gender dimorphism over the whole lifetime casts doubts on its direct relationship with the individual hormonal status. Thus, assessment of inflammatory cytokines in male and female TB subjects is needful because gender may be a major factor that influences the regulation of inflammatory factors; possibly due to a specific hormonal balance or adipose tissue distribution in women.^16^ Notably, an excessive accumulation of fat in the abdominal region, especially visceral obesity, was often associated with a prothrombotic and pro-inflammatory state.^17^

From the foregoing, sex differences in cytokines of tuberculosis infected subjects are expected, but the finding with anti-tuberculosis treatment is unclear. Anti-tuberculosis treatment regimen is divided into two phases of treatment namely; the intensive phase that includes the first 8 weeks of treatment with rifampicin, pyrazinamide isoniazid, ethambutol and the continuation phase of an additional 4 months (depending on treatment response) in which the subjects are given Rifampicin and Isoniazid.^18^ In this study therefore, the levels of TNF-α, IL-6, IL-2, IL-10 and TGF-β were measured in samples from freshly TB diagnosed male and female patients from whom samples were collected before treatment, after 2-months and 6-months of treatment to establish possible sex differences in levels of pro and anti-inflammatory cytokines before treatment and after each phase of therapy.

## Materials and methods

### Study design, area and setting

A comparative follow-up study was conducted at Mile Four Hospital Abakaliki, Ebonyi State. The hospital is found in Abakaliki town, located in South-Eastern part of Nigeria. Abakaliki is the state capital of Ebonyi State one of the five states located in the South-east geopolitical zone of Nigeria. It has Ibos as the dominant tribe. Mile Four hospital is a Catholic mission hospital that serves as a special referral centre for Tuberculosis in the state. As a result, a large number of people from various parts of the state visit the hospital both for inpatient and outpatient treatment. It has well equipped diagnostic laboratory for TB diagnosis and TB wards for in-patient admission of TB patients.

### Study Population

The study population was adult (male and female) smear and GeneXpert positive pulmonary tuberculosis patients who took all the intensive and continuation phase of treatment at the Tuberculosis clinic of Mile Four hospital during the study period. They received a 4 fixed dose combination of Rifampicin, Isoniazid, Pyrazinamide and Ethambutol hydrochloride for two months at the intensive phase of treatment and Rifampicin and Isoniazid only for 4 months at the continuation phase. In this study, TB patient less than 18 years of age, relapse cases, pregnant women, smokers, patient with other known clinical diseases such as cancer, HIV, chronic infections, organ impairments were excluded from this study.

### Sample size determination and sampling technique

A power analysis using G-power software (version 3.0.10) showed that a total sample size of 58 was needed to achieve a power of 90 at an alpha level of 0.05. But a total of 80 patients were recruited to give room for attrition in the course of follow-up of which only 60 patients (35 males and 25 females) were followed through to the sixth month (continuation phase therapy). This number was recruited by time delimited consecutive sampling technique in which all freshly diagnosed TB patients who were confirmed for tuberculosis infection and seeking for anti-tuberculosis treatment at Mile four hospital during the study period who meet the inclusion criteria were included.

### Ethical consideration

Ethical approval was obtained from the Ethics committee of Federal Teaching Hospital Abakaliki (FETHA) with reference number: FETHA/REC/VOL2/2018/105. A letter of permission was also obtained from the management of Mile Four hospital before the commencement of sample collection. The purpose, modalities and importance of the study was explained to the participants and verbal consent was obtained from each participant before recruitment into the study. To ensure confidentiality of participants in compliance with the Helsinki Declaration anonymity was maintained by ensuring that the name of the participant and any identifier of participants were not written on the questionnaire.

### Sputum sample collection and TB diagnosis

Two sputum samples (consisting of one spot sample and one early morning sample) were collected in a dry, clean, leak-proof, translucent, screw-capped, wide mouth plastic container from each subject. Tuberculosis diagnosis was carried out by both the Ziehl-Neelsen Acid fast bacilli (AFB) technique (in which smears were prepared and air dried and then stained with Ziehl-Neelsen stain and examined microscopically) as described by WHO 19as well as for the automated GeneXpert MTB/RIF real-time nucleic acid amplification test for rapid and simultaneous detection of TB and Rifampicin resistance as described by Blakemore *et al.* 20

### Blood sample collection

About four ml of venous blood was collected aseptically from each selected subject and dispensed into plain sample bottles labelled with a unique code number. The blood sample was collected from each study subject before initiation of anti-TB drugs, after completion of the two-month intensive phase and six-month continuation phase treatment. Serum was obtained after clotting by spinning at 3000rpm for 10 minutes and used for evaluation of TNF-α, IL-10, IL-6, IL-2 and TGF-β.

### Assay of inflammatory cytokines

Tumor necrosis factor - alpha (TNF-α), IL-10, IL-6, IL-2, transforming growth factor-beta (TGF-β) were assayed using ELISA test kits from U-CyTech Biosciences (Utrecht, Netherlands). The method employs quantitative sandwich enzyme immunoassay. A monoclonal antibody specific for each cytokine has been coated onto a microplate well. Subsequently, 100µl of blank, diluted standard, controls and samples were added to each well. The plates were sealed and incubated for two hours at 37^0^C and washed six times with the Wash buffer using the automated microplate, Washer. Then 100µl of diluted detection antibody solution was added to each well and the plate was sealed and incubated for 1 hour at 37^0^C. The washing step was repeated and 100µl of diluted SPP conjugate was added to each well and the plates sealed and incubated for one hour at 37^0^C. The washing step was repeated and 100µl of TMB substrate solution was added into each well and incubated in the dark for 20 minutes. The reaction was stopped with 100µl of Stop solution. Readings were taken using microplate reader at a wavelength of 450nm and the levels were quantified using reference standard curves.

### Statistical methods

The Statistical Package for Social Sciences (IBM SPSS Inc, Illinois, USA) version 22 was used in the statistical analysis. A normality test which was conducted to assess the distribution of each variable using Kolmogorov-Smirnov statistic showed that the data was not normally distributed. Thus, the data were expressed as Median (Range) in tables and Mann-Whitney test was used for comparison of the measured parameters between both gender (male and female) for each level of measurement. *P* values less than 0.05 were considered statistically significant.

## Results

At pre-treatment, the median IL-6 (pg/ml) was significantly higher in male TB subjects (81.59) compared to female TB subjects (55.59) (P=0.046) (Table 1).

**Table 1:** Comparison of median (range) levels of inflammatory cytokines between male and female TB patients at pre-treatment

Parameters	Male TB patients (n=35)	Female TB patients (n=25)	z-value	*P*-value
TNF-α (pg/ml)	1.55 (1.55-38.99)	1.55 (1.55-38.03)	-1.336	0.181
IL-6 (pg/ml)	81.59 (4.08-267.65)	55.59 (4.08-267.65)	-1.908	0.046*
IL-2 (pg/ml)	11.36 (11.36-15.36)	11.36 (11.36-14.36)	-0.570	0.569
IL-10 (pg/ml)	2.09 (1.46-10.39)	1.46 (1.46-10.39)	-0.970	0.332
TGF-β (pg/ml)	23.83(23.83-148.83)	23.83(23.83-123.83)	-1.194	0.232

* *P*<0.05 = Significant

TNF-α = Tumor necrosis factor alpha; IL-6 = Interleukin-6; IL-2 = Interleukin-2; IL-10 =Interleukin-10; TGF-β = Transforming growth factor beta

While after 2-months treatment, the median TNF-α (pg/ml) was significantly higher in males (100.11) compared with females (62.11) (P=0.008). Similarly, the median IL-10 (pg/ml) was significantly higher in males (5.27) compared with females (3.71) (P=0.045). Conversely, the median IL-6 (pg/ml) was significantly higher in female TB subjects (176.71) compared to the males (136.10) (P=0.042) (Table 2).

**Table 2:** Comparison of median (range) levels of inflammatory cytokines between male and female TB patients after intensive phase (2-months) treatment

Parameters	Male TB patients (n=35)	Female TB patients (n=25)	z-value	*P*-value
TNF-α (pg/ml)	100.11 (1.55-928.54)	62.11 (1.23-934.95)	-2.645	0.008*
IL-6 (pg/ml)	136.10 (4.08-259.47)	176.71 (4.08-241.19)	-1.072	0.042*
IL-2 (pg/ml)	16.98 (11.36-464.27)	12.09 (11.36-467.48)	-0.399	0.690
IL-10 (pg/ml)	5.27 (1.46-20.24)	3.71 (1.46-20.24)	-1.910	0.045*
TGF-β (pg/ml)	23.83(23.83-273.83)	23.83 (23.83-273.83)	-1.300	0.194

* *P*<0.05 = Significant

TNF-α = Tumor necrosis factor alpha; IL-6 = Interleukin-6; IL-2 = Interleukin-2; IL-10 =Interleukin-10; TGF-β = Transforming growth factor beta

However, after 6-months treatment, there was no significant difference in the median values of the inflammatory cytokines when compared between males and female TB subjects (P>0.05) (Table 3).

**Table 3:** Comparison of median (range) levels of inflammatory cytokines between male and female TB patients after continuation phase (6-months) treatment

Parameters	Male TB patients (n=35)	Female TB patients (n=25)	z-value	*P*-value
**TNF-α (pg/ml)**	15.31 (1.55-478.03)	28.91 (1.55-478.03)	-0.131	0.896
**IL-6 (pg/ml)**	74.06 (4.08-247.53)	74.30 (4.08-240.39)	-0.035	0.972
**IL-2 (pg/ml)**	11.36 (11.36-36.81)	11.36 (11.36-15.42)	-0.466	0.641
**IL-10 (pg/ml)**	2.21 (1.46-9.20)	2.90 (1.46-9.20)	-0.673	0.501
**TGF-β (pg/ml)**	198.83(23.83-5198.83)	148.83(23.83-5198.83)	-0.925	0.355

*P<0.05 = Significant

TNF-α = Tumor necrosis factor alpha; IL-6 = Interleukin-6; IL-2 = Interleukin-2

IL-10 =Interleukin-10; TGF-β = Transforming growth factor beta

## Discussion

In this study, following consecutive recruitment, there was a male preponderance, with TB-infected male to TB-infected female ratio of 1.4:1. This is similar to the findings of Nagpal *et al* 21 that obtained a male to female ratio of 1.5:1. This aligns with the fact that males are more susceptible to tuberculosis than females as reported in a previous study.^22^ Although the cause of this gender bias is not clearly defined, epidemiological factors have historically been considered the driving force.^3^ Males by virtue of their social behavioural pattern, may tend to travel more and spend time in crowded places such as bars, engage in mining and other professions that demands close physical contact. 4, 5 Also confounding factors, such as smoking, alcohol and drug use, which are known risk factors for TB, are more common in men 23, 24.

In this study, the median level of TNF-α and IL-10 did not differ between the male and female TB subjects before the initiation of treatment but after 2 months of treatment they were significantly higher in male TB subjects when compared with the female TB subjects. However, at the completion of the continuation phase therapy there was no obvious gender differences similar to what was obtained prior to the initiation of treatment. This implies that the two-months intensive phase therapy resulted in a significant increase in TNF-α and IL-10 levels in male TB subjects compared to female TB subjects. This confirms the generally observed trends of increased production of some cytokines in males compared with females.^11^ Our finding is supported by that of Cartier *et al* 25 as well as that of An *et al* 26 that tumor necrosis factor-alpha (TNF-α) concentrations was lower in women than in men which they attributed to the inhibitory effect of estrogen on the expression of genes that regulates the inflammatory markers. Even in animal study this finding holds true as reported by Inmaculada *et al* 27 that TNF-α were also suppressed in female compared with male mice. Aside from these there are also some contrasting research findings such as that by Wegner *et al* 12 that revealed greater pro-inflammatory responses in women compared with men in vivo. The observed increase in pro-inflammatory response was more significant in plasma TNF-α concentration. According to Popko *et al* 16 the higher levels in females could be due to the fact that both TNF-α and IL-6 are secreted by adipocytes which are more in females. Also, their concentration was shown to correlates with the percentage and distribution of fat tissue in the body which favours the female gender. However, Auda et al [28] found no significant difference between serum IL-10 concentration in male TB patients and female TB patients while Olaniyan *et al*^29^ found no significant gender and age influence on the plasma values of IL-10 and TNF alpha.

Moreover, the findings of this study shows that IL-6 level was significantly higher in males before treatment was initiated. Conversely, it was significantly higher in females after the intensive phase treatment, but there was no difference anymore after the continuation phase treatment. This implies that the intensive phase therapy resulted in a higher increase in the levels of IL-6 in females than the males which is a reversal of what was obtained before treatment, while at the completion of the continuation therapy there was no obvious gender differences in IL-6 level. Most studies align with our pre-treatment finding of a higher level of IL-6 in males than females. This includes the study by Cartier *et al*^25^ as well as that by Sperry *et al* 30 that found that IL-6 serum levels were statistically higher in males relative to females and this higher level of IL-6 expression in males remained statistically significant over time even after controlling for differences in age. This higher level of IL-6 in males may be possibly because of the inhibitory effect of estrogens on the expression of inflammatory marker genes in the females.^26^ However, some studies also support our findings of higher levels of IL-6 in females after the intensive phase therapy. Wegner *et al* 12 revealed greater in vivo pro-inflammatory responses in women compared with men, with significantly higher increases in plasma IL-6 concentrations. This finding could be due to the fact that markers of inflammation strongly correlate with measures of adiposity, and this association has been reported to be generally stronger in women than in men, especially for IL-6.^31^

## Conclusion

This study has identified that there are no gender differences in IL-2 and TGF-β irrespective of treatment status while gender differences exist in the levels IL-6, TNF-α and IL-10 at 2 months treatment and in IL-6 before treatment. Thus, anti-tuberculosis treatment contributes differentially to gender levels of these inflammatory cytokines in TB subjects.

The limitation of this study was that sex hormones were not assayed to establish the link between hormonal variations and gender differences in these parameters. Thus, a further study on the role of gender-based hormonal variations in the modulation of these parameters in TB subjects with therapy is recommended.
